# SABRE Hyperpolarization with up to 200 bar Parahydrogen in Standard and Quickly Removable Solvents

**DOI:** 10.3390/ijms24032465

**Published:** 2023-01-27

**Authors:** Anton Duchowny, Johannes Denninger, Lars Lohmann, Thomas Theis, Sören Lehmkuhl, Alina Adams

**Affiliations:** 1 Institut für Technische und Makromolekulare Chemie, RWTH Aachen University, 52074 Aachen, Germany; 2 Department of Chemistry, North Carolina State University, Raleigh, NC 27695, USA; 3 Institute of Microstructure Technology, Karlsruhe Institute of Technology, 76344 Eggenstein-Leopoldshafen, Germany

**Keywords:** high-pressure, benchtop NMR, SABRE, hyperpolarization, removable solvent

## Abstract

*Para*hydrogen (*p*-H_2_)-based techniques are known to drastically enhance NMR signals but are usually limited by *p*-H_2_ supply. This work reports *p*-H_2_-based SABRE hyperpolarization at *p*-H_2_ pressures of hundreds of bar, far beyond the typical ten bar currently reported in the literature. A recently designed high-pressure setup was utilized to compress *p*-H_2_ gas up to 200 bar. The measurements were conducted using a sapphire high-pressure NMR tube and a 43 MHz benchtop NMR spectrometer. In standard methanol solutions, it could be shown that the signal intensities increased with pressure until they eventually reached a plateau. A polarization of about 2%, equal to a molar polarization of 1.2 mmol L^−1^, could be achieved for the sample with the highest substrate concentration. While the signal plateaued, the H_2_ solubility increased linearly with pressure from 1 to 200 bar, indicating that *p*-H_2_ availability is not the limiting factor in signal enhancement beyond a certain pressure, depending on sample composition. Furthermore, the possibility of using liquefied ethane and compressed CO_2_ as removable solvents for hyperpolarization was demonstrated. The use of high pressures together with quickly removable organic/non-organic solvents represents an important breakthrough in the field of hyperpolarization, advancing SABRE as a promising tool for materials science, biophysics, and molecular imaging.

## 1. Introduction

**Figure 1:**
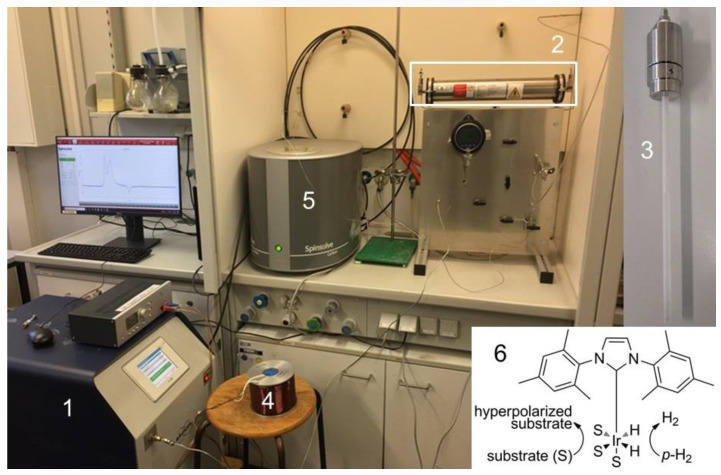
Overview of the high-pressure *p*-H_2_ SABRE setup: *para*-hydrogen generator (1), high-pressure cylinder (2), high-pressure sapphire tube (not shown to scale for a better view) (3), electromagnet (4), benchtop NMR device (5), along with the SABRE polarization transfer mechanism in the iridium-based catalyst employed in this study (6).

Although nuclear magnetic resonance (NMR) techniques are among the most important analytical methods for obtaining detailed information about materials on the molecular level, they suffer from inherently low sensitivity. Even when examining protons, which are ubiquitously present in organic molecules, only a tiny fraction effectively contributes to the detectable signals under thermal equilibrium conditions in standard NMR spectrometers. This low sensitivity can lead to lengthy measurement times. Stronger magnetic fields incrementally increase the spin polarization but are associated with exponentially increasing costs. Even at magnetic fields of ~24 T in modern GHz NMR spectrometers, proton spin polarization is only on the order of 10^−4^. Alternatively, polarizations approaching unity, associated with dramatic signal enhancements, can be obtained with hyperpolarization techniques. Important examples include dynamic nuclear polarization (DNP) [1,2] and *para*-hydrogen (*p*-H_2_) induced polarization (PHIP), including signal amplification by reversible exchange (SABRE) [3,4,5]. SABRE is based on the reversible association of *p*-H_2_ and a substrate with an iridium catalyst [6] (Figure 1(6)) and is currently a method of high interest as it provides a simple, low-cost route for signal enhancement. In addition, the enhanced NMR signals can be regenerated easily, for example, by simply re-shaking the samples or by resupplying new *p*-H_2_ via bubbling or with the help of continuous flow reactors [7,8,9,10].

Many factors influence the achievable SABRE hyperpolarization. They include exchange rates at the catalyst [11,12,13,14,15,16,17], the *p*-H_2_ supply (i.e., flow, bubbling, and shaking), the solubility [12,14,16,18,19,20], and the concentration [5,11,12,15,21,22] of the catalyst, hydrogen, and substrate in a chosen solvent. Further important parameters include the presence of co-substrates or additives [12,23,24,25] and the exact control of the polarization transfer field [11,12,13,14,15,17,26,27,28,29]. Another critical parameter that may often limit the hyperpolarization is the *p*-H_2_ pressure applied to the sample [11,12,13,14,26,28,30,31,32]. The often-encountered linear dependence between applied pressure and signal enhancement suggests that further signal enhancements are possible with increasing pressures. Yet, despite the development of dedicated high-pressure *p*-H_2_ generators [33,34,35,36,37], so far, SABRE hyperpolarization experiments have been performed below 10 bar of pressure. This limitation is probably due to the lack of a suitable setup for *p*-H_2_ supply at high pressures and a lack of low-cost, commercially available high-pressure NMR tubes. An easy-to-replicate and inexpensive high-pressure tube design was reported recently [38]. Moreover, a recent report has described a low-cost and versatile high-pressure setup employing a high-pressure sapphire tube, allowing spectroscopic analyses of samples pressurized up to 200 bar with a benchtop NMR spectrometer. It has been applied successfully to study compressed gases as well as gas–liquid and gas–solid interactions [39,40].

This work introduces the aforementioned high-pressure setup for *p*-H_2_ experiments as a simple way to overcome the current pressure limitations. The experimental setup is depicted in Figure 1, with more details provided in Section 4. Instead of exposing the sample to the *p*-H_2_ gas directly from the standard *p*-H_2_ generator, Figure 1(1), the *p*-H_2_ gas is redirected into a piston cylinder, Figure 1(2), which can also serve as a mixing reservoir. This detour allows for quick and easy pressure adjustments, independent of the maximum 7–10 bar output pressure of the *p*-H_2_ generator. The pressurized *p*-H_2_ alone, or in a mixture with another gas, is loaded into the high-pressure sapphire tube, Figure 1(3), containing the evacuated sample (substrate, catalyst, and possibly solvent). The loaded sapphire tube is physically connected to the high-pressure cylinder during the entire hyperpolarization experiment. First, the tube is shaken for 10 s, then it is inserted into a sinusoidal electromagnet, Figure 1(4), set to produce a magnetic field of 69 G for another 10 s, finally followed by insertion into the benchtop NMR equipment, Figure 1(5), for immediate measurement. 

Three classes of experiments were performed using this setup. First, the ingress of hydrogen gas in deuterated methanol up to 200 bar is monitored to identify a possible solubility limit. In our study, a linear relationship without a solubility limitation of hydrogen gas was observed. Secondly, the first pyrazine SABRE hyperpolarization results with up to 200 bar *p*-H_2_ pressure measured in a 43 MHz benchtop NMR spectrometer are reported. Finally, since the high-pressure setup contains a mixing reservoir, alternative solvents for the substrate/catalyst were tested as a proof-of-concept. For instance, ethane liquefaction in an ethane/*p*-H_2_ mixture was exploited, similar to a different study [41]. However, in [41], propane was hyperpolarized by hydrogenating propene, whereas here, ethane merely serves as the carrier substance. Similarly, a CO_2_/*p*-H_2_ mixture was brought onto a solid catalyst–pyrazine mix free of solvent. In this case, the compressed CO_2_ liquefied the sample and dissolved *p*-H_2_, leading to signal enhancement. 

The presented results open new avenues for low-cost and versatile high-pressure hyperpolarization and help to understand the effect of *p*-H_2_ pressure on signal enhancement, especially with respect to achieving high polarization at high substrate concentrations, i.e., high molar polarization. This work also provides a path to easily remove organic solvents, resulting in more biocompatible injectables better suited for biomolecular imaging and medical research. Removal of organic solvents is crucial before SABRE hyperpolarization can be efficiently deployed in vivo. The introduced methodology is also expected to play a significant role in analyzing trace substances and complex mixtures and in material characterization [12,22]. Moreover, the structure and dynamics of biopolymers may be studied, which are known to be affected by the application of pressure [42,43,44]. 

## 2. Results

### 2.1. Dissolution of Hydrogen in Deuterated Methanol

**Figure 2:**
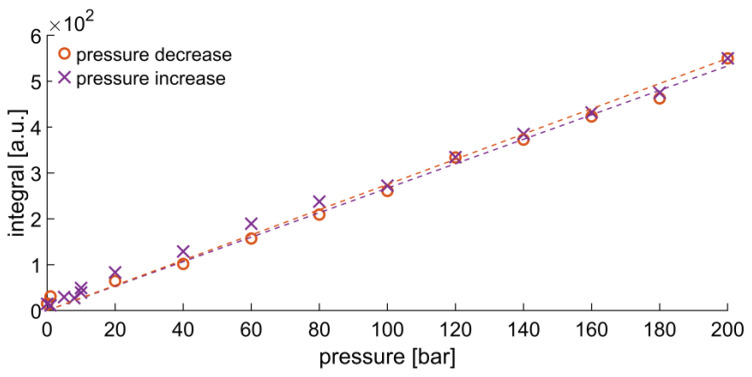
Peak integral of H_2_ dissolved in *d*-methanol between 1 and 200 bar. Only the right half of the H_2_ peak was integrated to reduce the influence of the overlapping OH peak from residual non-deuterated methanol. The dotted lines are linear fits of H_2_ peak integrals obtained when raising the pressure from 0 to 200 bar (purple) or lowering it from 200 to 1 bar (orange).

The dissolution of H_2_ gas in deuterated methanol *(d*-methanol, CD_3_OD) between 1 and 200 bar was examined to prove the absence of a solubility limit in the analyzed pressure range. A linear dependence can be seen for increasing and decreasing the H_2_ pressure, as demonstrated in Figure 2. In the investigated pressure range, no solubility limit was detected. Hence, increasing the H_2_ pressure results in an increased H_2_ concentration in the sample solution. Although deuterated methanol was utilized, the mole fraction of dissolved hydrogen could not be precisely quantified due to peak overlap. Nevertheless, the observed linear trend agrees with the published literature data [45,46,47,48]. The spectra were acquired a few seconds after pressure application, resembling the hyperpolarization experiments. Therefore, no temporal effects of H_2_ mixing or diffusion into the solvent need to be considered for data evaluation.

### 2.2. High-Pressure SABRE Hyperpolarization of Pyrazine in d-Methanol 

**Figure 3:**
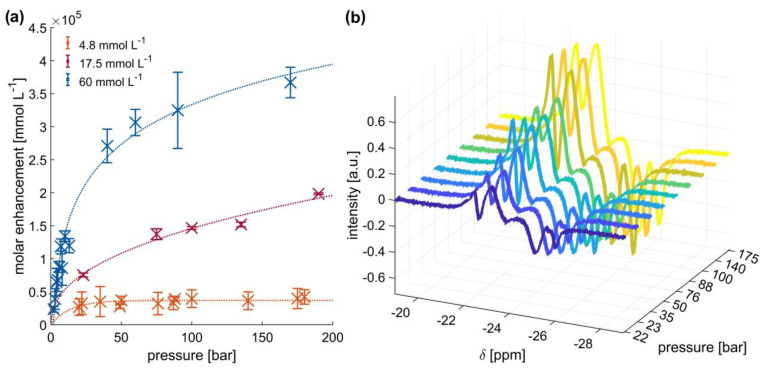
(**a**) Molar enhancements achieved with high-pressure SABRE hyperpolarization at different pyrazine concentrations in *d*-methanol. The pyrazine/catalyst ratio was 1/20. The thermal signal of a reference sample was used for determining the signal enhancement. The dotted lines serve as a visual guide. The measurement for each pressure point was conducted three times to determine the error bars. (**b**) Hydride signals of hyperpolarized solutions with 4.8 mmol L^−1^ pyrazine and 0.24 mmol L^−1^ catalyst at different pressures.

Pyrazine, with its four chemically and magnetically equivalent protons, gives a single line in the proton NMR spectrum; thus, pyrazine was chosen as a model substrate to investigate the effect of pressure at different substrate concentrations on the SABRE signal enhancement. The effect of increasing the *p*-H_2_ pressure on the molar signal enhancement (= enhancement × concentration, directly proportional to total signal) of this substrate is depicted in Figure 3a. First, a sample with 60 mmol L^−1^ pyrazine in *d*-methanol was investigated. The catalyst/substrate ratio was 1/20 and this ratio was kept constant for all investigated samples. While a quasi-linear signal increase was observed at lower pressures, as already demonstrated by other studies [48,49], this is not the case beyond 10 bar. Instead, the signal gains level off. Nonetheless, a 3-fold enhancement was achieved by increasing the pressure from 10 to 200 bar. Moreover, for this 60 mmol L^−1^ sample, we achieved an enhancement of around 6 × 10^3^ with a corresponding molar enhancement of 3.6 × 10^5^ mmol L^−1^ (6 × 10^3^ × 60 mmol L^−1^), which is equal to a polarization of about 2%. This corresponds to a molar polarization of 1.2 mmol L^−1^. However, we note that experimental reproducibility was associated with relatively large error bars, as depicted in Figure 3a. Utilizing an automated transfer shuttle from the polarization transfer magnet to the NMR magnet for detection would significantly improve the experimental reproducibility [7]. 

While a similar non-linear behavior with pressure was also detected for the 17.5 mmol L^−1^ sample, a constant signal enhancement was obtained with the lowest concentrated sample (4.8 mmol L^−1^) above 20 bar. A similar finding was reported in PHIP experiments with decreasing substrate concentration [22]. This finding is consistent with theoretical predictions [49], where at lower substrate concentrations, the achievable enhancements and hyperpolarization levels become limited by factors other than the *p*-H_2_ concentration. Hence, lower *p*-H_2_ pressures would be sufficient to conduct experiments with low substrate concentrations. 

Furthermore, the effect of increasing *p*-H_2_ pressure on the hydride signals of the Ir catalyst was investigated. As exemplarily shown for the 4.8 mmol L^−1^ sample in *d*-methanol in Figure 3b, an increasing hydride signal was observed with increasing pressure, even though the pyrazine signal enhancement remained constant. This outcome illustrates that *p*-H_2_ was successfully brought into the sample and it is not the limiting factor for higher enhancements. Similar behavior was observed for the hydrides of the other two concentrations, as illustrated for the 17.5 mmol L^−1^ sample in Figure S1 of the Supplementary Information. Figure S2 shows that with increasing pressure, there is continued increase in the hydride signals in all samples. Moreover, the hydride spectra show no signs of a structural change in the catalytic complex with increasing *p*-H_2_ pressure. Thus, it can be assumed that the hyperpolarization mechanism is mostly independent of pressure in the sense that the active hyperpolarization transfer species does not change. 

In summary, the results further substantiate that: (a) *p*-H_2_ can be brought into the solution efficiently at high pressures, (b) the polarization transfer mechanism seems to stay the same at elevated pressures, and (c) the observed enhancements at low pyrazine concentrations level off and are not limited by *p*-H_2_ availability at elevated pressures. However, higher *p*-H_2_ pressures generate a significant boost in signal enhancement for higher substrate concentrations (d). Such an enhancement boost is important, for example, when aiming for a maximum molar polarization for hyperpolarized MRI contrast agents. 

### 2.3. High-Pressure SABRE Hyperpolarization with Quickly Removable Solvents

Methanol is a solvent not suited for medical applications because of its toxicity. However, it is typically the solvent of choice for SABRE because of its reduced hydrogen solubility and non-optimal exchange kinetics in water. Here, we provide proof-of-concept experiments that replace methanol with removable gaseous solvents that are only liquefied at sufficiently high pressures. 

In the first experiment, the liquefaction of ethane as a SABRE solvent was investigated. For this purpose, a 50/50 mixture comprising ethane and *p*-H_2_ at 12 bar was produced in the piston cylinder of the high-pressure setup. Subsequently, the mixture was compressed to 200 bar total pressure. Up to ~40 bar partial pressure at ambient temperature, ethane is gaseous and mixes with the *p*-H_2_ gas. Once the partial pressure exceeds this value, ethane liquefies, thus obtaining a liquid *p*-H_2_/ethane system that can be brought into the sample. Upon decreasing the pressure, both *p*-H_2_ and ethane can evaporate. Although this approach is promising in theory, it was found that liquefied ethane did not dissolve the catalyst nor pyrazine, and hence, pyridine as a substrate was chosen instead and the same catalyst was supplied in a *d*-methanol solution (catalyst/pyridine 1/20 molar ratio). 

**Figure 4:**
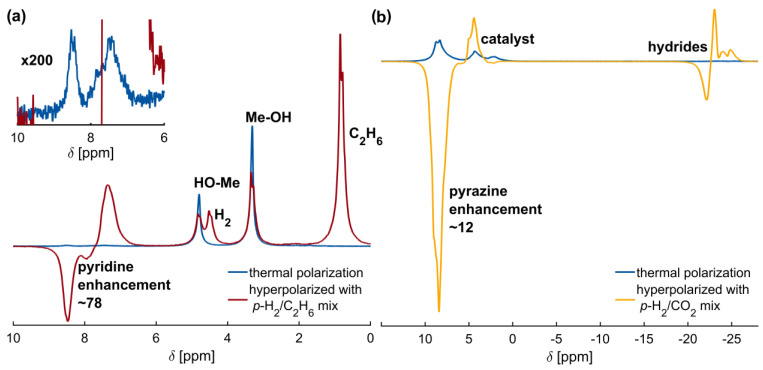
Parahydrogen hyperpolarization in quickly removable solvents. (**a**) ^1^H NMR spectrum of pyridine in *d*-methanol hyperpolarized by liquefying a *p*-H_2_/ethane mixture with a signal enhancement of about 78 (red). The insert shows a 200-fold magnification of the thermal pyridine signal. The polarization transfer field for this experiment is the earth’s magnetic field. (**b**) Hyperpolarization of solid pyrazine and catalyst with a pressurized *p*-H_2_/CO_2_ 50:50 mixture at 200 bar. The achieved signal enhancement is about 12. Reference thermal polarization spectra are depicted in blue.

The feasibility of this approach was confirmed by the experimental data showing hyperpolarized pyridine peaks, illustrated in Figure 4a. Comparing the peak’s signal-to-noise levels before and after hyperpolarization yields an enhancement factor of at least 78. This enhancement factor is a lower bound of the actual value because the hyperpolarized sample is partially carried out of the spectrometer’s sensitive volume upon releasing the gas. Accordingly, exact quantification was not possible in this first feasibility study, yet with certainty, we can maintain that large enhancements in excess of seventy-eight were achieved.

In a second proof-of-concept experiment, CO_2_ was used to avoid methanol completely. CO_2_ is considered a green solvent with great potential to replace organic solvents because it is inexpensive, non-toxic, environmentally more acceptable, and non-flammable [50,51]. It is a suitable solvent for pyrazine at moderate temperatures and pressures, even when not supercritical [52]. The required conditions to dissolve pyrazine in CO_2_ at room temperature can easily be achieved when operating the presented experimental setup at higher pressures. As in the previous experiment, gaseous CO_2_ was mixed with *p*-H_2_ in the piston cylinder, compressed to 200 bar total pressure, and filled into the NMR tube. This time, the sample comprised only the solid catalyst and pyrazine (1:20 molar ratio) without any *d*-methanol (Figure S3). The compressed CO_2_ liquefied the solids (Figure S4) and transferred the *p*-H_2_ to the catalyst and analyte, as demonstrated by the hyperpolarized signal shown in Figure 4b. However, only a small droplet was formed in the sapphire tube that partially filled the sensitive volume. The unevenly filled sensitive volume led to a broad linewidth and lower signal-to-noise ratio. The observed signal enhancement, in this case, was around twelve compared to the spectrum acquired at thermal equilibrium (pressurized and liquefied sample in CO_2_).

Additionally, the hydrides between −20 and −30 ppm were strongly enhanced in the hyperpolarized spectrum and have a similar signal strength to the hydrides from samples dissolved in *d*-methanol (Figure S5). However, the spectral signatures differ in appearance due to the formation of different complexes [53,54]. After releasing the gas, the liquefied chemicals recrystallized again (Figure S3), and no signal could be detected. This is due to the solid-state dipolar broadening in the crystalline solid, which could not be removed with the used liquid probe head. The observation of a comparable hydride signal strength in CO_2_ and *d*-methanol, yet much lower pyrazine enhancement, indicates that the polarization transfer to the substrate is inefficient in CO_2_. Possible reasons include the different polarity of CO_2_ compared to methanol, possible conformation changes of the catalyst and/or substrate in the presence of CO_2_, and a diminished lifetime of the catalytic SABRE complex, not allowing enough time for the polarization transfer. Yet, given the strong hydride peaks, strong binding and insufficient exchange of pyrazine at the catalytic complex is the most likely explanation. By resolving some of these possible limitations, it can be expected that significant further signal enhancement will become possible by fine-tuning the experimental conditions.

## 3. Materials and Methods

The high-pressure setup used in this work has been described in the literature previously [40]. In brief, gas was filled into a piston cylinder with two physically separated compartments. Turning a 3-way valve redirected the sample gas to the high-pressure NMR tube containing the sample solution. By applying pressure to the other compartment of the piston cylinder, a movable piston compressed the sample gas until the pressure was equilibrated. Additionally, the setup was equipped with several safety features. A safety valve releases gas when a pressure above 206 bar is reached and is attached to the high-pressure line connecting to the piston-cylinder, since this line will always be pressurized. Pressure ratings for all other parts are beyond 400 bar. Furthermore, check valves were installed after the gas inlet ports, preventing highly pressurized gas from flowing back into the gas bottle. 

The employed NMR tube was made of sapphire, capable of withstanding high pressures. However, it is brittle and susceptible to lateral forces from bending or impacts. Hence, special care must be taken when performing the experiments, especially while shaking and inserting the tube into the polarization transfer or NMR magnet. A spacer made of foam was attached below the high-pressure tube attachment to soften the impact of inserting the pressurized tube into the NMR magnet. This spacer also ensured that the sample was always in the same region of the sensitive volume. Likewise, the electromagnet had a foam insert, which guaranteed that samples were always exposed to the same magnetic field and reduced the chance of hitting the tube. Employing an automated sample transferring shuttle, such as in Ref. [7], could greatly improve the safety aspect of the high-pressure experimental setup.

A *p*-H_2_ generator (Bruker BioSpin GmbH, Karlsruhe, Germany) was attached at the sample gas inlet port, filling the piston-cylinder with up to 7 bar of *p*-H_2_ (Figure 1). The proton NMR experiments were recorded using a 1 T ^1^H-frequency benchtop spectrometer (Magritek GmbH, Aachen, Germany) working at a proton frequency of 43 MHz. For the hyperpolarization experiments conducted below 8 bar, the generator was connected directly to the high-pressure tube. 

For the experiments including gaseous solvents, *p*-H_2_ and ethane or CO_2_ must be filled successively into the piston cylinder. After filling *p*-H_2_, the piston cylinder’s valve was closed and the *p*-H_2_ generator was exchanged with the ethane or CO_2_ gas bottle. After flushing and evacuating the high-pressure lines multiple times with ethane or CO_2_, the second gas component was filled into the piston cylinder until the desired mixing ratio was reached. From this point on, the experimental procedure is the same as above.

Pyrazine and pyridine were purchased from Sigma-Aldrich (Merck KGaA, Darmstadt, Germany) and methanol-*d*_4_ from Eurisotop (Cambridge Isotope Laboratories Inc., Tewksbury, MA, USA). These chemicals were dried and degassed thoroughly before usage. The iridium-based catalyst was synthesized in-house according to Ref. [26], stored under an argon atmosphere, and shielded from sunlight. Samples with 60, 17.5, and 4.8 mmol L^−1^ pyrazine were prepared with a 1/20 catalyst/pyrazine molar ratio. The same molar ratio of 1/20 was also used for the catalyst/pyridine sample. H_2_ 5.0 (99.999% purity), CO_2_ 4.5 (99.995% purity), and ethane 3.5 (99.95% purity) were purchased from Westfalen AG (Münster, Germany) and used without further purification. 

## 4. Conclusions

SABRE hyperpolarization experiments at pressures up to 200 bar are reported. A simple, low-cost, and versatile high-pressure setup was attached to a standard commercially available *p*-H_2_ generator to exceed its maximum achievable pressure. The proposed experimental setup can be a viable alternative to the current state-of-the-art setups for generating pressurized *p*-H_2_. 

The applicability of this novel approach was demonstrated by hyperpolarizing pyrazine with an iridium-based catalyst dissolved in *d*-methanol in a high-pressure NMR tube. The presented results demonstrated that pressures beyond the current maximum of ~10 bar allow further signal enhancement, albeit not following the quasi-linear trend observed at low pressures. It was shown that the higher the substrate concentration, the higher the molar polarization. In contrast to the substrate signals, the hydride signal intensity increased steadily with pressure, which shows that this polarization can potentially be better harnessed by changing other experimental parameters, such as temperature and sample composition. 

Furthermore, the versatile design of the high-pressure setup allowed applying ethane and CO_2_ as alternative and easily removable solvents in the hyperpolarization experiments. Highly pressurized CO_2_ liquefied the solid catalyst and substrate, in which *p*-H_2_ subsequently dissolved, and thus a signal enhancement could be detected. In contrast, ethane could neither dissolve the catalyst nor pyrazine. However, the feasibility of exploiting the ethane liquefaction at high pressure was demonstrated with pyridine and might be useful in different hyperpolarization substrates and settings. Eliminating standard SABRE organic solvents such as methanol in hyperpolarization techniques is a prerequisite for molecular medical research. Even if the practical approach to high-pressure SABRE hyperpolarization needs to be fine-tuned, this work highlights that it is possible to overcome current experimental limitations.

## Data Availability

Not appliable.

## Supplementary Materials

The corresponding supporting information can be downloaded at: https://www.mdpi.com/article/10.3390/ijms24032465/s1.
