# Supplementary material for: SABRE Hyperpolarization with up to 200 bar Parahydrogen in Standard and Quickly Removable Solvents
Source: Int J Mol Sci. 2023 Jan 27;24(3):2465. doi: 10.3390/ijms24032465 (PMC9917027; doi:10.3390/ijms24032465)
Supplement: Supplementary file 1 [file ijms-24-02465-s001.zip › ijms-2154083-supplementary.pdf]

International Journal of
Molecular Sciences

MDPI

Communication

Supplementary Information for

SABRE hyperpolarization with up to 200 bar parahydrogen in standard and quickly removable solvents

Anton Duchowny[1], Johannes Denninger[1], Lars Lohmann[1], Thomas Theis[2], Sören Lehmkuhl[3], and Alina Adams[1*]

[1] Institut für Technische und Makromolekulare Chemie, RWTH Aachen University, 52074 Aachen, Germany
[2] Department of Chemistry, North Carolina State University, Raleigh, North Carolina 27695, USA
[3] Institute of Microstructure Technology, Karlsruhe Institute of Technology, 76344 Eggenstein-Leopoldshafen, Germany
* Correspondence: Alina.Adams@itmc.rwth-aachen.de

Table of contents

Figure S1: Hydride signals of hyperpolarized solutions with 17.5 mmol L^{-1} pyrazine at different pressures.

Figure S2: Integrals of hydride signals with increasing pressure. The dotted lines serve as a visual guide.

Figure S3: Photo-collage showing the solid catalyst-pyrazine sample before applying *p*-H_2/CO_2 (left and middle) and the recrystallized sample after removing the gas (right).

Figure S4: Photo-collage showing the liquefied catalyst-pyrazine sample after applying gas pressure. The top left and bottom photos show the sample pressurized at 200 bar with only CO_2 to acquire the spectrum at thermal polarization. The upper right photo shows the activated catalyst under *p*-H_2/CO_2.

Figure S5: Hydride signals of hyperpolarized pyrazine solutions with two solvents: *d*-methanol (green) and in liquefied CO_2 (blue).

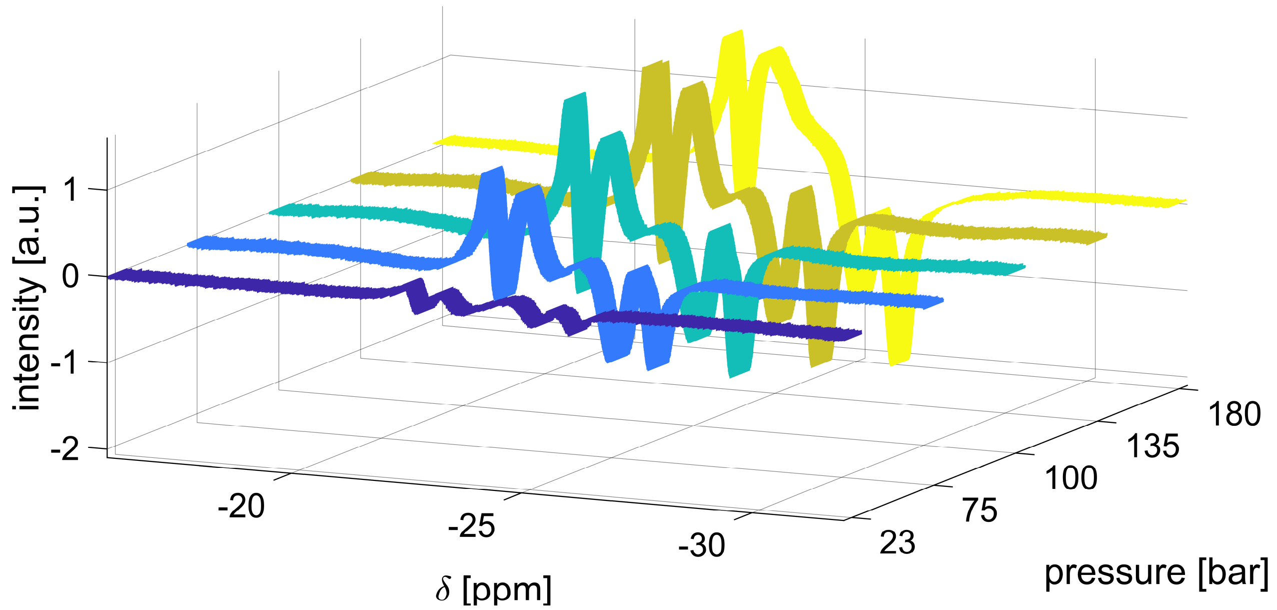

Figure S1: Hydride signals of hyperpolarized solutions with 17.5 mmol L-1 pyrazine at different pressures.

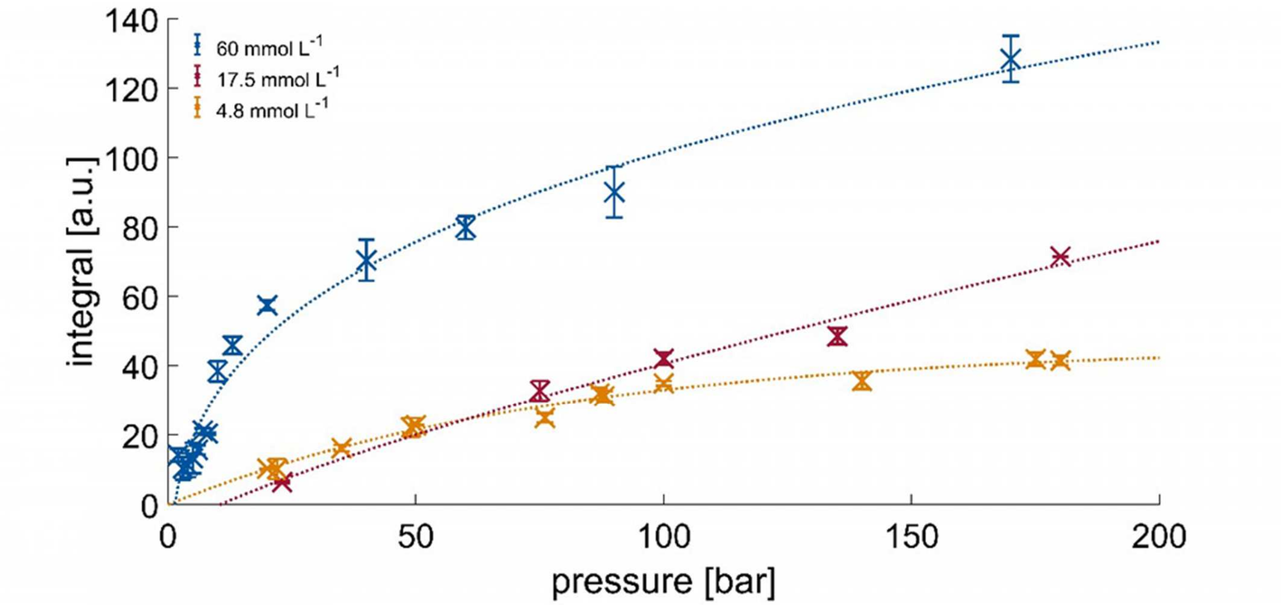

Figure S2: Integrals of hydride signals with increasing the pressure. The dotted lines serve as a visual guide.

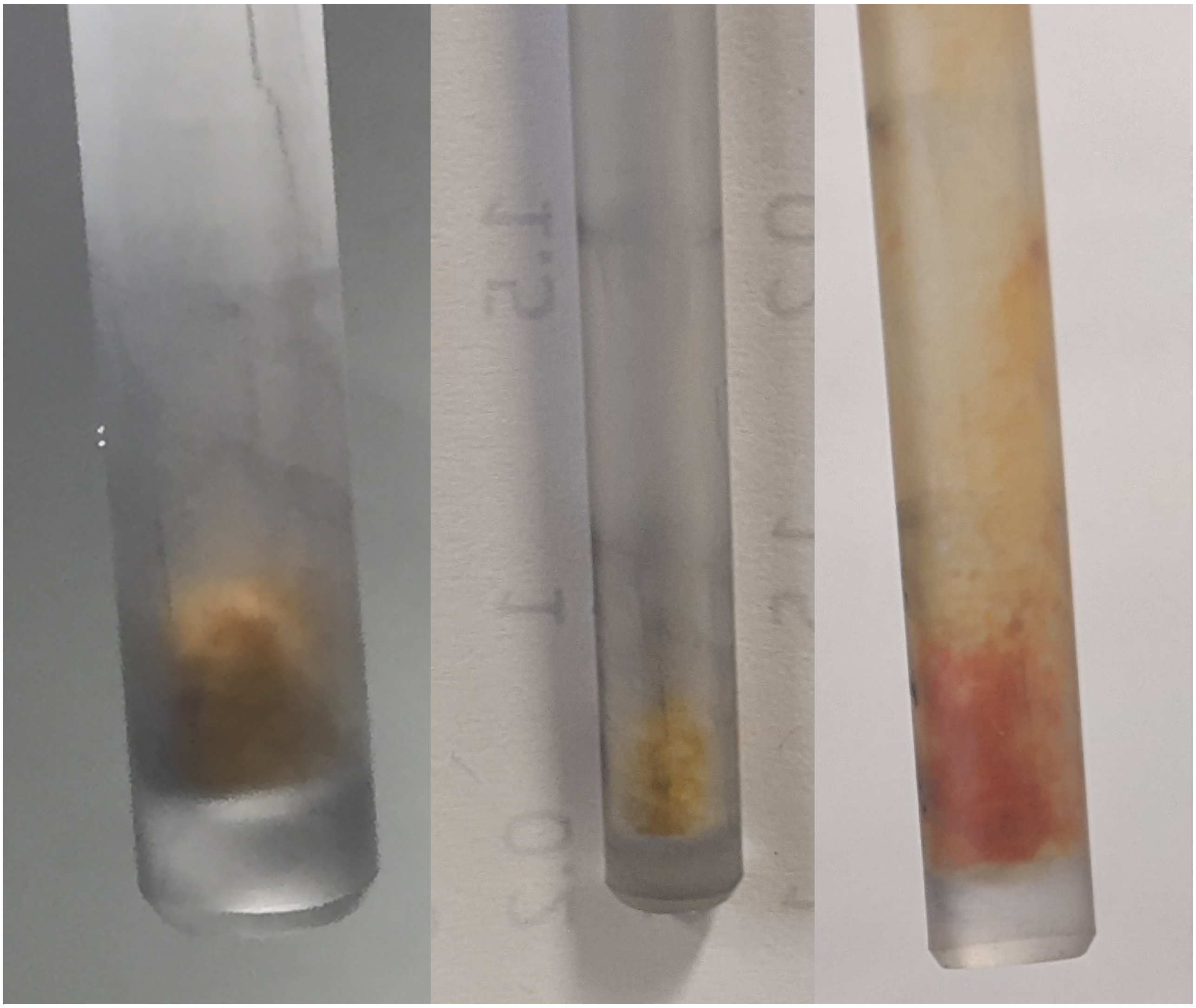

Figure S3: Photo-collage showing the solid catalyst-pyrazine sample before applying *p*-H_2/CO_2 (left and middle) and the recrystallized sample after removing the gas (right).

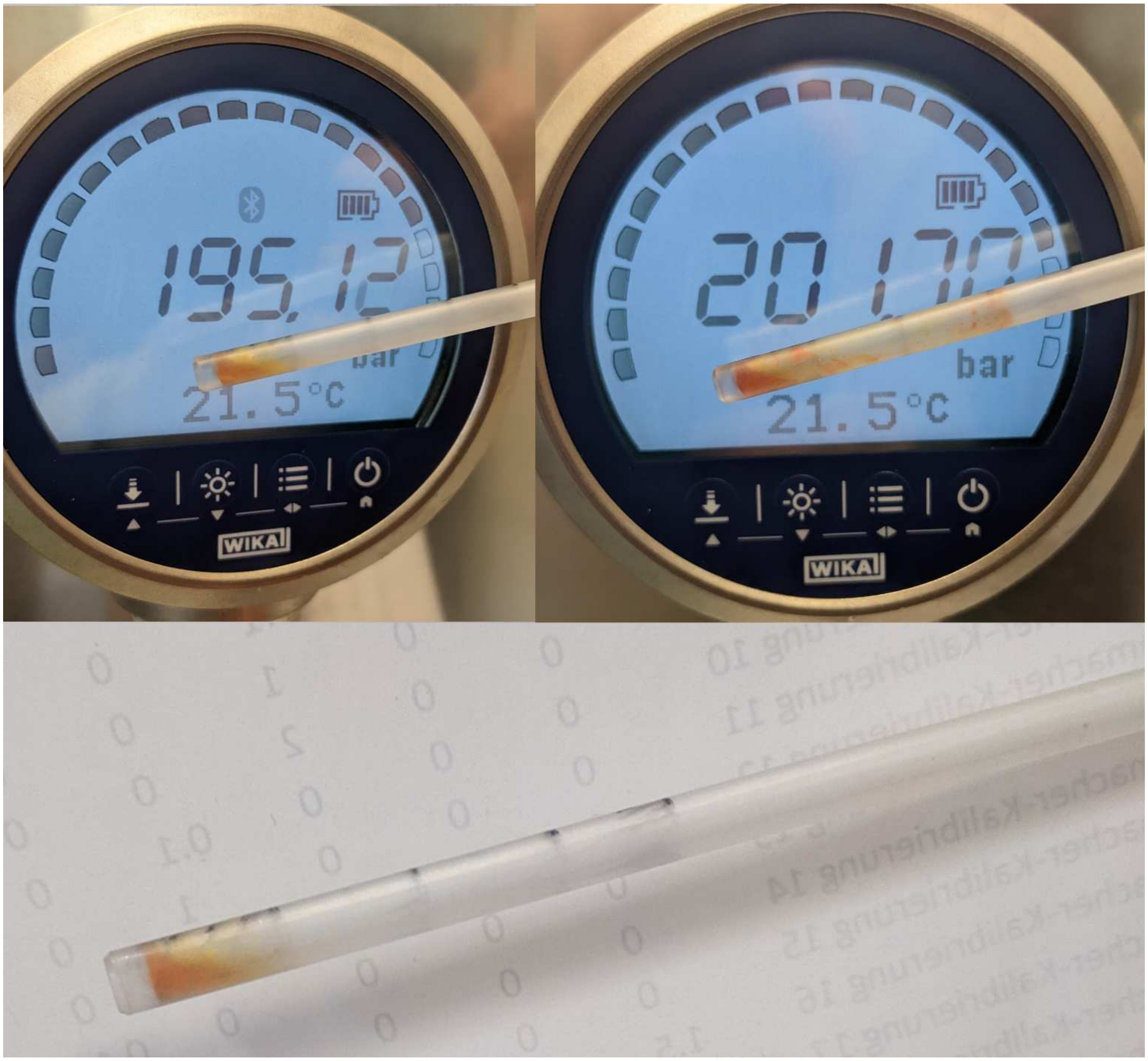

Figure S4: Photo-collage showing the liquefied catalyst-pyrazine sample after applying gas pressure. The top left and bottom photos show the sample pressurized at 200 bar with only CO_2 to acquire the spectrum at thermal polarization. The upper right photo shows the activated catalyst under *p*-H_2/CO_2.

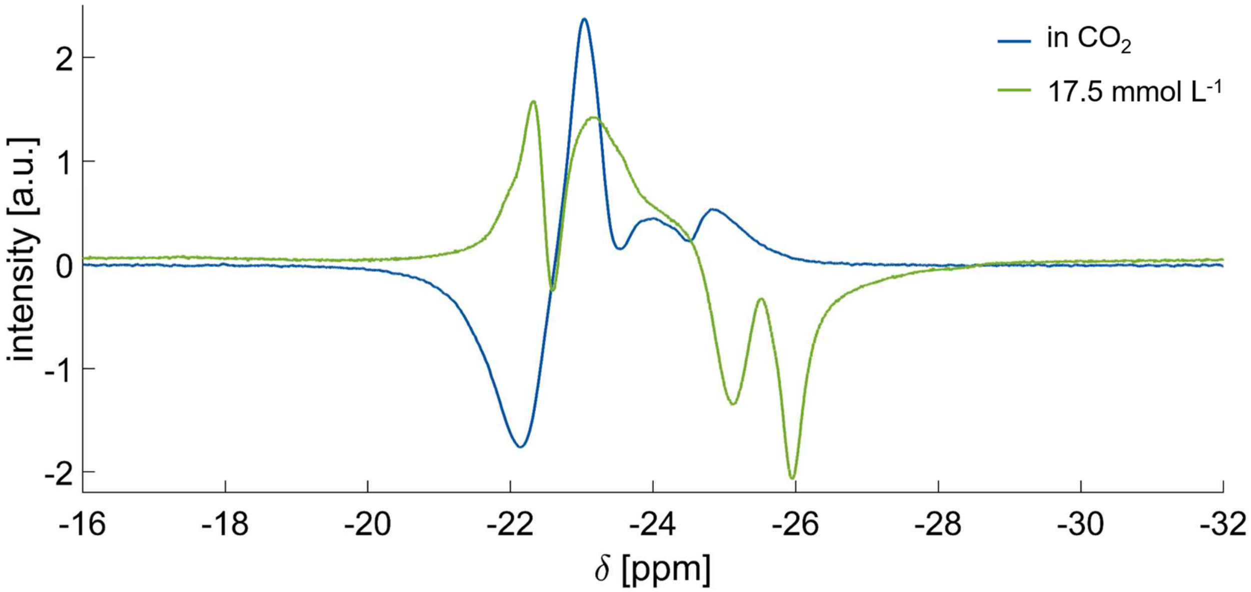

Figure S5: Hydride signals of hyperpolarized pyrazine solutions with two solvents: *d*-methanol (green) and in liquefied CO_2 (blue).
